# Outcomes following mepolizumab treatment discontinuation: real-world experience from an open-label trial

**DOI:** 10.1186/s13223-019-0348-z

**Published:** 2019-06-10

**Authors:** Hector Ortega, Catherine Lemiere, Jean-Pierre Llanos, Mark Forshag, Robert Price, Frank Albers, Steven Yancey, Mario Castro

**Affiliations:** 1 Respiratory US Medical Affairs, GlaxoSmithKline, La Jolla, CA USA; 2 Faculty of Pharmacy, Université de Montréal, Montreal, QC Canada; 3 Research Centre, Hôpital du Sacré-Cœur de Montréal, Montreal, QC Canada; 4 Respiratory US Medical Affairs, GlaxoSmithKline, Research Triangle Park, NC USA; 5 Clinical Statistics, GlaxoSmithKline, Stockley Park, Uxbridge, Middlesex, UK; 6 Respiratory Therapeutic Area Unit, GlaxoSmithKline, Research Triangle Park, NC USA; 7 Division of Pulmonary and Critical Care Medicine, Washington University School of Medicine, St. Louis, MO USA; 8 Present Address: Gossamer Bio, 3013 Science Park Rd, Suite 200, San Diego, CA 92121 USA

**Keywords:** Severe eosinophilic asthma, Cessation of treatment, Asthma control, Blood eosinophils

## Abstract

Limited information is available on the clinical course of patients with severe asthma following discontinuation of biologic treatment. Therefore, a post hoc analysis was conducted in patients with severe eosinophilic asthma who participated in the COSMOS trial, where patients received mepolizumab for more than 1 year of continuous therapy. The objective of this post hoc analysis was to evaluate changes in the Asthma Control Questionnaire (ACQ-5) and blood eosinophil counts 12 weeks after the last administration of mepolizumab. Cessation of mepolizumab was associated with a rise in the blood eosinophil count and loss of asthma control after stopping therapy. These data suggest that patients with severe disease require extended and continuous treatment. Further studies evaluating longer duration of continuous treatment with mepolizumab could help understanding of whether changes in the presentation of the disease (disease modification) are possible with the use of biologics, such as mepolizumab.

**To the Editor:**

## Background

Asthma is a heterogeneous disease with diverse characteristics and biologic mechanisms. Current asthma guidelines offer a helpful framework for managing patients; however, some patients remain uncontrolled despite aggressive treatment interventions. The major goal of these guidelines is to achieve disease control and reduce the risk of future deterioration [1, 2]. Although the guidelines do discuss the management of patients with severe asthma, they were not designed for a phenotype or endotype-driven approach to care. Personalized treatment interventions for Th2-high asthma do not benefit all individuals since not all patients have Th2-high disease. Stratification of asthma subtypes (i.e., phenotypes and endotypes) with appropriate use of biomarkers can help patient selection and guide management. The use of biomarkers (both clinical and lab-based) that are easily measured and consistently reliable is essential. In the case of severe eosinophilic asthma, the use of blood eosinophils as a biomarker to select the patients most likely to benefit with anti-interleukin 5 (anti-IL5) therapies has been established [3–5]. Similarly, clinical markers of uncontrolled asthma including a recent history of exacerbations despite optimized treatment, or uncontrolled asthma based on asthma control (e.g., ACQ or ACT) are relevant tools in the assessment of these patients. Recently, several biologics have been approved for the treatment of patients with severe asthma. However, there are critical questions when a patient with severe asthma initiates treatment with a biologic, such as the length of treatment duration and potential consequences after stopping treatment. The current report evaluated changes that occurred following mepolizumab (anti-IL5 monoclonal antibody) therapy cessation after more than 1 year of continuous therapy.

## Methods

COSMOS [6] was a 52-week, multicenter, open-label, phase IIIb study that assessed the safety of mepolizumab 100 mg subcutaneous (SC) in patients (N = 651) with severe eosinophilic asthma (NCT01842607). Eligible patients were ≥ 12 years of age, who upon completion of randomized studies MENSA [3] (NCT01691521) or SIRIUS [4] (NCT01691508) immediately commenced the COSMOS trial. Mepolizumab 100 mg SC was administered every 4 weeks with the last dose administered at week 48. At week 60, patients (57%) who did not immediately enter a subsequent open-label extension (COSMEX, Study ID 201312) returned to clinic for a follow-up visit. The subsequent COSMEX study was designed to enroll patients with the most severe form of asthma (as identified by previous intubations, hospitalizations, exacerbations and maintenance oral corticosteroids use) in patients who had previously demonstrated benefit from mepolizumab treatment. This approach ensured that mepolizumab treatment was available for an extended period for those with the greatest unmet medical need.

In the current analysis, ACQ-5 and blood eosinophil counts were chosen as their deterioration may predict subsequent asthma worsening as well as exacerbations and increases in oral corticosteroid dose. ACQ-5 and blood eosinophils were measured at weeks 4, 16, 28, 40 (ACQ-5 only), 52 (exit visit; 4 weeks after the last administration of mepolizumab), and 60 (follow-up). Values of the endpoints measured are presented as means with 95% confidence intervals (CI) to show measure of spread, in addition to presenting the proportion of subjects above and below an ACQ-5 score and blood eosinophil count of 1.5 and 150 cells/µL, respectively.

## Results

**Fig. 1:**
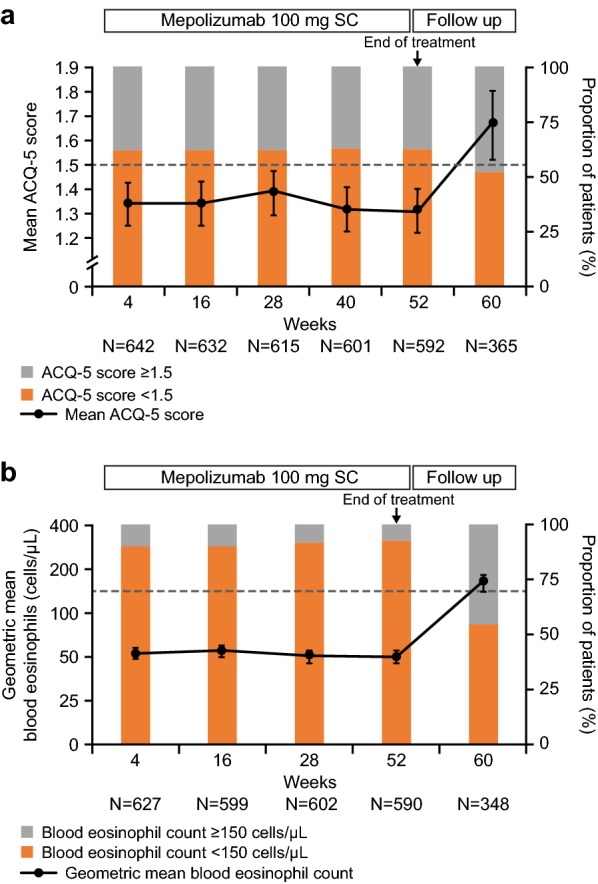
**a** Mean ACQ-5 scores, **b** geometric mean blood eosinophil counts including proportions of subjects with mean ACQ-5 score ≥ 1.5/< 1.5 and blood eosinophil counts ≥ 150/< 150 cells/µL. Mean ACQ-5 scores and geometric blood eosinophil counts were measured during the open label period and following treatment discontinuation. Error bars represent 95% confidence interval. Reference line for **a** at a mean ACQ-5 score of 1.5. Reference line for **b** at a geometric mean blood eosinophil count of 150 cells/µL. *ACQ-5* Asthma Control Questionnaire-5, *SC* subcutaneous

Upon completion of mepolizumab treatment (week 52) in the COSMOS study, the mean ACQ-5 score was 1.31 (95% CI 1.22–1.40). ACQ-5 measured 12 weeks after the last administration of mepolizumab (week 60) showed a reduction in asthma control (movement in ACQ-5 values to ≥ 1.5, reflective of uncontrolled asthma) with a mean ACQ-5 score of 1.66 (95% CI 1.52–1.80) (Fig. 1a). For patients completing both the exit visit and the follow-up visit (weeks 52 and 60), ACQ-5 mean score increased, indicating a worsening in asthma symptoms, from 1.28 (95% CI 1.16–1.40; n = 326) at week 52 to 1.65 (95% CI 1.51–1.79; n = 357) at week 60. Overall, of the 592 patients with data available, almost two-thirds (n = 372) had an ACQ-5 score below the 1.5 threshold at week 52, and by week 60 this had decreased to about half (188/365).

As reported previously by Lugogo et al. [6], the geometric mean of blood eosinophils was 48 cells/µL (95% CI 45–52) at week 52. Following cessation of mepolizumab treatment, eosinophil counts measured at week 60 increased to a geometric mean of 159 cells/µL (95% CI 141–179) (Fig. 1b). For patients completing both the exit visit and the follow-up visit (weeks 52 and 60), blood eosinophil counts (geometric mean) also increased from 45 cells/µL (95% CI 40–49; n = 327) at week 52 to 158 cells/μL (95% CI 140–178; n = 338) at week 60. Overall, for blood eosinophil counts at week 52, 91% of patients (536/590) had a count below 150 cells/µL, which decreased to 43% (149/348) by week 60.

## Discussion

The increase in the mean ACQ-5 score (0.35 points) 8 weeks after treatment cessation, although not considered clinically significant, suggests that patients began to experience a worsening of asthma control only a few weeks following cessation of mepolizumab treatment. In addition, at 12 weeks after the last administration of mepolizumab (week 60) the mean ACQ-5 scores were similar to the values reported at baseline, demonstrating that when treatment with mepolizumab was discontinued patients’ asthma symptoms showed signs of worsening and in parallel blood eosinophils increased. The threshold of 150 cells/µL has previously been associated with the responder phenotype to mepolizumab treatment [3–5].

Our findings are consistent with a previous study by Haldar et al. [7] in patients with severe eosinophilic asthma (N = 56) who experienced a significant increase in blood eosinophil counts after discontinuation of mepolizumab and a subsequent clinical deterioration, i.e., at 12 months following discontinuation of mepolizumab, the mean modified Juniper Asthma Control Questionnaire score increased by 0.59 points to an estimated score of 2.29 (p < 0.001) [7]. This study by Haldar and colleagues also addressed the question regarding rebound, which is defined as an exaggerated pharmacodynamic response following treatment cessation above baseline values. In their study as well as in the current analysis, the return of symptoms and mean blood eosinophil counts were not seen to exceed baseline values at the start of treatment.

A limitation of the current study was the lack of a control arm, and thus between-study comparisons should be made with caution. The fact that more severe patients entered the COSMEX extension study could have introduced some selection bias in those subjects attending the follow-up visit. However, consistent results were observed following analyses of the overall study population and when restricted to only subjects completing both the exit visit and the follow-up visit (weeks 52 and 60). Furthermore, mean values of the ACQ-5 and blood eosinophil data at the end of the COSMOS study (week 52) were similar to baseline values in those patients who entered the COSMEX study with continuous treatment. In addition, we were unable to extend our observations beyond 12 weeks after the last administration of mepolizumab and therefore it is unknown if a further deterioration occurred in these patients. Another limitation is that the dose of other controller medications was not regulated throughout the study period. As such, it is difficult to establish comparisons with controlled clinical trials in which controller medication use is regulated. However, this lack of controller use regulation should be considered to reflect the real-world clinical experience of patients receiving long-term mepolizumab treatment. Overall, these data highlight the importance for continuous treatment with a biologic based on changes in key clinical outcomes after discontinuation of mepolizumab treatment.

## Data Availability

Anonymized individual participant data from the studies listed within this publication and their associated documents can be requested for further research from www.clinicalstudydatarequest.com.

## Abbreviations

ACQ-5: Asthma Control Questionnaire-5
ACT: Asthma Control Test
anti-IL5: anti-interleukin-5
CI: confidence interval
SC: subcutaneous
